# Prevalence of common thrombophilia markers and risk factors in Indian patients with primary venous thrombosis

**DOI:** 10.1590/S1516-31802010000500004

**Published:** 2010-09-02

**Authors:** Mahendra Narain Mishra, Varinder Singh Bedi

**Affiliations:** I MD. Specialist in Pathology and Immunopathology, Department of Pathology, Command Hospital, Southern Command, Pune, Maharashtra, India.; II MS. Professor and Head of the Department of Vascular Surgery, Army Hospital Research & Referral, Delhi Cantt, India.

**Keywords:** Asian continental ancestry group, Venous thrombosis, Risk factors, Thrombophilia, Antibodies, anticardiolipin, Grupo com ancestrais do continente asiático, Trombose venosa, Fatores de risco, Trombofilia, Anticorpos anticardiolipina

## Abstract

**CONTEXT AND OBJECTIVE:**

Venous thrombosis occurs as a result of interaction of genetic and acquired factors including activated protein C resistance (APC-R), fibrinogen levels, antithrombin, protein C, protein S, lupus anticoagulants and anticardiolipin antibodies. This study was aimed at determining the prevalence of these common thrombophilia markers in Asian Indians with primary venous thrombosis.

**DESIGN AND SETTING:**

This was a cross-sectional study carried out in Mumbai.

**METHODS:**

Samples from 78 patients with a confirmed diagnosis of venous thrombosis and 50 controls were tested. Semi-quantitative estimation (functional assays) of protein C, protein S and antithrombin was performed. Quantitative estimation of fibrinogen was done using the Clauss method. Lupus anticoagulants were screened using lupus-sensitive activated partial thromboplastin time and β2-glycoprotein-I dependent anticardiolipin antibodies were estimated by ELISA. APC-R was measured using a clotting-based method with factor V deficient plasma and *Crotalus viridis* venom. Statistical analysis was performed using Epi-info (version 6).

**RESULTS:**

The popliteal vein was the most commonly involved site. Forty-four samples (56%) gave abnormal results. The commonest were elevated fibrinogen and APC-R (17.9% each), followed by low protein S (16.6%).

**CONCLUSIONS:**

This study confirms the literature findings that fibrinogen level estimation and screening for APC-R are important for the work-up on venous thrombosis patients since these, singly or in combination, may lead to a primary thrombotic episode. The frequency of the other thrombophilia markers was higher among the patients than among the controls, but without statistically significant difference.

## RESUMO

**CONTEXTO E OBJETIVO:**

A trombose venosa ocorre como resultado da interação de fatores genéticos e adquiridos, incluindo resistência à proteína C ativada (APC-R), os níveis de fibrinogênio, antitrombina, proteína C, proteína S, anticoagulante lúpico e anticorpos anticardiolipina. Este estudo teve como objetivo verificar a prevalência de fatores trombofílicos frequentes em indianos com trombose venosa primária.

**TIPO DE ESTUDO E LOCAL:**

Estudo transversal analítico realizado em Mumbai.

**MÉTODOS:**

Foram testadas amostras de 78 pacientes com diagnóstico confirmado de trombose venosa e 50 controles. Foi realizada a dosagem sérica semiquantitativa (funcional) de proteína C, proteína S e antitrombina e a dosagem quantitativa de fibrinogênio (método de Clauss). Anticoagulantes lúpicos foram identificados por meio do tempo de tromboplastina parcial ativada sensível ao lúpus, e anticorpos anticardiolipina dependentes de β2-glycoproteína-I por ELISA. APC-R foi medida por método baseado em coagulação com plasma deficiente em fator V e veneno de *Crotalus viridis*. A análise estatística utilizou Epi-info (versão 6).

**RESULTADOS:**

A veia poplítea foi o local mais frequentemente afetado; 44 amostras (56%) tiveram resultados anormais. Os achados mais frequentes foram elevação do fibrinogênio e APC-R (17,9% cada), e baixa proteína S (16,6%).

**CONCLUSÕES:**

Corroborando com a literatura, este estudo mostrou que a elevação do nível de fibrinogênio e a triagem para APC-R são importantes na avaliação de pacientes com trombose venosa, pois, individualmente ou em combinação, podem ter levado ao episódio trombótico primário. A frequência dos outros marcadores de trombofilia foi mais alta entre os doentes quando comparados aos controles, porém sem diferença estatisticamente significante.

## INTRODUCTION

Venous thrombosis occurs due to interaction of several genetic and acquired factors.^1^ The genetic factors include activated protein C resistance (APC-R), deficiency of anticoagulant proteins such as antithrombin (AT), protein C and protein S. Antiphospholipid antibodies (APL) are the commonest cause of acquired thrombophilia, among which lupus anticoagulants (LA) and β2 glycoprotein I dependent anticardiolipin antibodies (aCL) are clinically the most relevant. There have been very few studies on the incidence among Asian Indians. The contemporary Indian literature of the last four years on thrombophilia was studied and significant variations were found in the frequency of common thrombophilia markers.^2-5^ Since the cost of thrombophilia investigations is quite phenomenal, for a developing country like India, prudent selection of relevant tests is of utmost importance.

## OBJECTIVE

This study was carried out to study the prevalence of seven common thrombophilia markers including LA, aCL, APC-R, AT, protein C, protein S and fibrinogen in an Asian Indian population with venous thrombosis, compared with controls without venous thrombosis.

## METHODS

This was a cross-sectional analytical study performed at the Indian Naval Hospital Ship Asvini, Colaba, Mumbai, Maharashtra, India. Sixty-three consecutive patients with venous thrombosis who were admitted to this 800-bed superspeciality hospital between October 2001 and November 2003 were the subjects of this study. In addition, data pertaining to 15 patients treated elsewhere, but followed up at a 500-bed government hospital between February 2004 and October 2006 were analyzed. Five patients had a history of both venous and arterial thrombosis.

The control population comprised 50 age and sex-matched voluntary blood donors and asymptomatic volunteers. It is desirable for all laboratories performing thrombophilia screening to have their own standards. Therefore, we tested a control population of fifty volunteers so that we could have our own laboratory standards. The patients and controls were matched for gender and age.

The inclusion criteria for the patients were that they should present one or more of the following: (i) age < 45 years at onset of thrombotic episode; (ii) absence of any obvious cause that may have led to the thrombotic episode; (iii) more than one episode of thrombosis irrespective of age; and (iv) thrombosis at unusual sites. Ten patients were more than 45 years of age at the time of initial presentation but were included because they had multiple episodes of thrombosis at unusual sites.

The diagnostic methods for detecting thrombosis were ultrasonography or venography for deep vein thrombosis, and radio nucleotide lung scanning or angiography for pulmonary embolism, with the use of computed tomography scans and magnetic resonance imaging where necessary.

Informed consent from all participants and approval from the Ethics Committee were obtained for this study.

### Sample collection and processing

Samples were collected from all patients 10-12 weeks after the acute episode. Coumarins were discontinued and the patients were administered low molecular weight heparin for three weeks before drawing the sample. Venous blood was drawn into blue Vacutainer tubes (Becton Dickinson, New Jersey, United States) containing 0.105 M sodium citrate (nine volumes of blood to one volume of anticoagulant) and centrifuged at 2500 g for 15 minutes at room temperature and again at 4 °C to remove platelets. The supernatant plasma was stored at -35 °C in 0.2 ml aliquots until testing. At the time of testing, frozen plasma samples were thawed directly in a 37 °C water bath for at least 15 minutes and then mixed thoroughly by swirling before use. The assay calibrations were performed automatically using a barcoded STA-Unicalibrator (Diagnostica Stago, France). The laboratory control values for diagnosing thrombophilia markers were derived by testing the control population. The range was found to be similar to what is mentioned in the manufacturer’s reference material, except for protein C and protein S, which were slightly higher (Table 1).

**Table 1.** Range and mean values for controls and their comparison with the manufacturers’ references

Test	Control range n = 50	Mean	Range/mean (product reference)
PT	11.1 - 13.2 s	12.1	11.5 - 14.5
APTT	26 - 34 s	29	32.4
LA-PTT	27 - 40 s	34	37.4
AT	79 - 124%	97	80 - 120
Protein C	89 - 150%	105	70 - 130
Protein S	91 - 150%	110	65 - 140
APC-R	120 ≥ 240 s	> 120	> 120 s
Fibrinogen	0.13 - 0.30 g/l	0.18	0.2 - 0.4
aCL IgG	1.1 - 8 GPL units/l	4	< 10
aCL IgM	2 - 5 MPL units/l	3	< 7

PT = prothrombin time; APTT = activated partial thromboplastin time; LA-PTT = lupus-sensitive activated partial thromboplastin time; AT = antithrombin; APC-R = activated protein C resistance; aCL IgG = anticardiolipin antibody of immunoglobulin G isotype; aCL IgM = anticardiolipin antibody of immunoglobulin M isotype; s = seconds; l = liter; g/l = gram per l; GPL = immunoglobulin G phospholipid units/l; MPL = immunoglobulin M phospholipid units/l.

### Equipment

Clotting-based tests were done using Start 4 (Diagnostica Stago, France), which is a four-channel semiautomatic coagulometer. This was calibrated using commercial calibrators prior to opening each new batch of kits. Complete blood counts (CBC) were done using a hematology cell counter; enzyme-linked immunosorbent assay (ELISA)-based testing for aCL was done using a semiautomatic ELISA reader (Labsystems, United States). AT estimation was carried out using the RA-50 apparatus (Bayer, United States).

### Methods

Any histories of important risk factors, including smoking, hyperlipidemia, diabetes mellitus and positive family histories of thrombosis, were elicited from all patients and controls. All the patients had normal hepatic function.

The initial tests included complete blood count, prothrombin time, activated partial thromboplastin time (APTT), lupus sensitive APTT (LA-PTT) and fibrinogen levels. The biochemical tests included blood sugar, liver function tests, serum cholesterol and lipid profile. Screening for LA was done by means of LA-PTT and positive results were confirmed by failure to correct prolonged APTT in mixing studies using normal plasma. Functional assays on thrombophilia markers were performed in duplicate by means of clotting-based methods. APCR testing was done in the presence of Factor V deficient plasma, using an APTT-based test. All tests were carried out in accordance with the product inserts and manual. Tests were performed in batches of 20-25 samples, including frozen normal samples as well as commercial controls consisting of normal (N) and abnormal (N+P) plasma. Fibrinogen levels were tested by means of the Clauss method.^6^ Functional assays on protein C were performed using a modified APTT on citrated patient plasma, which was also added to protein C deficient plasma and then activated with *Agkistrodon c. contortrix* venom.

Clotting was begun by the addition of 0.025 M calcium chloride. APC, produced by the venom, inhibited factors V and VIII (provided by the protein C deficient plasma) and thus prolonged the clotting time obtained by means of addition of calcium chloride. A standardized amount of APC was added to diluted plasma samples for protein S estimation. Activation of endogenous protein C was performed after the clotting time had been determined, and after simultaneous coagulation activation via the intrinsic system using an APTT reagent, using tissue factor Va. AT estimation involved a synthetic peptide that mimicked the natural target substrate of the enzyme and was attached to a chromogenic group at or near the cleavage site. In this assay, patient plasma was incubated with an excess of thrombin, in the presence of heparin. In the first phase of the reaction, the antithrombin neutralized the thrombin in the presence of heparin. The remaining thrombin, which was inversely proportional to the amount of antithrombin in the patient plasma, was then quantified according to the cleavage of para-nitroaniline from the peptide substrate at 405 nm. The levels of protein C, protein S and AT in test plasma were expressed as percentages (%) of the standard plasma. Plasma in which the clotting time was greater than or equal to 120 seconds was considered to be APC-R negative. Confirmation of a positive result for aCL or LA was given only after testing a second sample drawn at least 6-8 weeks later, in accordance with the "Sapporo" laboratory criteria.^7^

### Statistical analysis

Statistical analyses on the accrued data were done using the Epi Info (version 6) software. The chi-square test was used for calculation of P values. The Yates and Mantel-Haenszel corrections were applied to the data. Fisher’s exact test (both one and two-tailed) was performed and its results were used for drawing conclusions.

Sample size was calculated considering a study power of 60%, odds ratio worth detecting of 1.00, exposure of APC-R among controls of 6% and a 90% confidence interval, resulting in a minimum sample of 53 patients.

Thrombophilia markers were labeled as positive only after confirmation on another fresh sample drawn at least two months later. This was to make the criteria for labeling patients as positive for thrombophilia markers more stringent, and because antigenic assays were not done in this study.

## RESULTS

A total of 78 patients (65 males and 13 females) and 50 age-matched controls (42 males and eight females) were studied. Multiple episodes of thrombosis occurred in 12 patients. No abnormality was detected in 34 samples, while 44 samples (56.4%) were positive for one or more thrombophilia markers, of which 14 (18%) had more than one thrombophilia marker. One patient had four thrombophilia markers, including raised fibrinogen, protein S deficiency, APC-R and aCL. Protein S deficiency was present in 13 patients, of whom three patients also had APC-R. In one of these, the father was also deficient in protein S.

The mean age and range for patients and controls were 36.5 years (21-55 years) and 34 years (25-45 years) respectively. Four patients gave family histories of protein S deficiency; however, screening of family members was beyond the scope of this study. The commonest clinical presentation was deep vein thrombosis of lower limbs (n = 63), and the popliteal vein was the commonest site. Table 2 shows the thrombosis sites and clinical presentations of 15 patients; Table 3 shows the frequencies of thrombophilia markers and risk factors in patients and controls, along with the low or peak abnormal values, as relevant. The mean fibrinogen levels in the controls and patient population were 0.183 and 0.334 g/l respectively, with a peak level of 0.798 g/dl in patients. Low AT levels were detected in five patients, of whom three were only marginally low (60-65%, while the lower limit of the normal range was 70%).

**Table 2.** Clinical presentation and sites of thrombosis other than popliteal vein

Presentation (cases)	Age/sex	Abnormalities	Site of involvement
Small bowel ischemia(2)	31/M	Protein S low	Mesenteric vein
	35/F	Proteins C and S low Fibrinogen raised	Mesenteric vein
Portal hypertension (2)	39/M	Protein S low	Portal vein in both cases
	40/M	Proteins C and S low	
Avascular necrosis of femoral head (2)	43/F	Protein S low, APC-R.	Femoral (left)
	48/M	APC-R, high fibrinogen	Popliteal and femoral veins (both)
Post-delivery (1)	24/F	Protein S low, APC-R	Internal iliac vein
Post-MTP (1)	26/F	LA	Internal iliac vein
Sudden blindness, multiple episodes (2)	50/F	AT low	Branch retinal vein
	60/F	Low protein S	Central retinal vein
Other sites (5)	23/F	LA	Inferior vena cava
	44/F	LA, AT, Proteins C an S low	Inferior vena cava
	44/F	Protein S low	Axillary vein: 1
	38/F	Protein S low	Jugular vein: 1
	32/M	NAD, smoker	Subclavian veins: 1

F = female; M = male; MTP = Medical termination of pregnancy; AT = antithrombin; NAD = no abnormality detected; APC-R = activated protein C resistance; LA = lupus anticoagulants.

**Table 3.** Prevalence of thrombophilia markers and risk factors among patients: semi-quantitative and P values

No.	Thrombophilia marker/risk factor	Number of controls (n = 50)	Number of patients (%)	Abnormal values (quantitative)	P value
1	No abnormality	44	34 (43.6)	–	–
2	Protein S	1	13 (16.6)	13-47.5%	0.001
3	Protein C	0	6 (7.7)	10-60%	0.019
4	Antithrombin	0	5 (6.4)	44-65%	0.124
5	APC-R	2	14 (17.9)	NQ	0.042
6	LA	2	7 (9)	NQ	0.402
7	ACL (IgG)	2	4 (5.1)	23-140 GPL	0.56
8	Fibrinogen	0	14 (17.9)	504-798 mg/dl	0.026
9	Smoking	5	7 (9)	NQ	0.846
10	Dyslipidemia	2	5 (6.4)	NQ	0.086
11	Family history	0	4 (5.1)	NQ	0.06

NQ = not a quantitative parameter; LA = lupus anticoagulants; ACL (IgG) = anticardiolipin antibody (immunoglobulin G isotype); mg/dl = milligrams per deciliter; APC-R = activated protein C resistance; GPL = immunoglobulin G phospholipid units/l.

### Statistically significant parameters

The P values for APC-R, low protein S and protein C, raised fibrinogen and a positive family history were statistically significant while the prevalence of ACL, LA, smoking, dyslipidemia and low AT were higher in the patients but were not significantly related to deep vein thrombosis in this study.

## DISCUSSION

The timing of sample collection and interpretation of tests is of utmost importance. False low values of protein C, protein S and AT are known to occur during the acute phase, and samples should be tested only 8-12 weeks after stabilization. Coumarins and APL are also known to cause artifactually low values; therefore, the samples must be taken only after the patients have been weaned off coumarins and have been administered low molecular weight heparins for three weeks. If APL is present, false APC-R and low protein S may be detected. Functional protein S estimates are unreliable in the presence of APCR, and the current recommendation is to screen with antigen assays.^8^ In such cases, if available, antigenic assays on protein S and DNA analysis for factor V Leiden become imperative. However, these are not routinely available and are more expensive. Simultaneous APL and APC-R/low protein S were present in only two patients and this was also confirmed in fresh samples from the patients. Therefore, this finding was probably not artifactual.

The incidence of Factor V Leiden is reported to be low in Indian patients with thrombosis, ranging from 3 to 4.1%.^4,5^ On the other hand, surprisingly, APC-R has been reported in 12.5 to 39.2% of Indian patients.^2,3^ In this study, APC-R was detected in 19% of the patients and 4% of the controls. The method used is supposed to have over 99% sensitivity and specificity for factor V Leiden.^8-10^ One limitation of this study was that molecular studies were not done for factor V Leiden, which is the commonest cause of APC-R. Thus, there is a need to look for other mutations that could lead to APC-R in Indians.

Lupus anticoagulants and anticardiolipin antibodies were present in 9% and 5% of the patients, respectively. The raised anticardiolipin antibodies were all of immunoglobulin G (IgG) isotype and were in the range of 21-30 immunoglobulin G phospholipid (GPL) units/l in three out of four patients, while the value of the remaining sample was 140 GPL units/l. From this, it can be inferred that the immunoglobulin M (IgM) isotype is probably not of etiological importance for venous thrombosis. Many studies have expressed anticardiolipin antibodies as GPL and immunoglobulin M phospholipid (MPL) units/l, such that one unit is equivalent to one gram of immunoglobulin G (IgG) (for GPL) or IgM (for MPL) anticardiolipin antibodies, purified from 1 ml of serum.^11^

Our rate of positive APL findings was similar to what has been reported in the contemporary Indian literature. Some other Indian workers have found only moderate increases in aCL levels in Indian populations, and high values are rare.^10^ The presence of aCL and LA in various Indian studies has been reported to be 5.3 to 21% and 6.25%, respectively.^3,4,12^ Some authors have described concordance rates for positive LA and aCL results ranging from 60 to 90%,^13^ but in this study none of the ten positive samples showed such concordance. Therefore, we are of the opinion that all samples must be tested for both aCL and LA. Raised fibrinogen levels were present in 18% of the cases, and higher levels correlated with recurrence and severity of thrombotic episodes.

The frequencies of low antithrombin, protein C and S in this study were higher than what has been reported in some Indian studies.^3,4^ Bhattacharyya et al.^2^ reported protein S, protein C and AT deficiencies in 8.5, 9.7 and 1.6% of patients without additional risk factors. The incidence of protein S deficiency in Western studies ranges from 0.8 to 3.2%^14,15^ and, when combined with protein C and antithrombin, these account for 5-10% of the cases.^15^ It is quite possible that our higher results were due to the testing method and also that some of these were not due to inherited deficiency, since family studies were not performed. 60% of the patients with low AT levels only showed marginal decreases (60-65%). In the normal range (70-130%), only moderate decreases in AT have been reported to be associated with thrombosis.^16-18^ The overall much higher frequency of protein S deficiency (16.6%), which was four times greater than in Western studies^14,15^ could be partly attributed to reductions in C4b protein, as a result of higher rates of subclinical infections among Indians. In none of the cases was an underlying cause present; therefore, most of them were considered to consist of inherited deficiencies of anticoagulant proteins. This, along with not testing for protein S by means of antigenic assays is a weakness of this paper. Protein C and AT are preferentially tested by functional assays alone.

All the samples were collected from 63 consecutive patients 10-12 weeks after the acute episode. The sample size was calculated using the assumption that the power of this study (beta) was 60%, odds ratios worth detecting were 1.00, the percentage exposure among controls regarding APC-R was 6% and the confidence interval was 90%. With a ratio of controls to samples of one, the minimum sample size was 53 for controls and patients. In this study, the total sample size was 128. This study was also limited by financial allocations. One limitation of this study was the small sample size of 78 patients, which gave a power of only 60%, rather than the ideal of 80%. Secondly, we only performed functional assays and not antigenic assays for AT, protein C and protein S. Factor V Leiden was not included in this study, and this would have confirmed whether patients with APCR had the mutation.

The strengths of this study include the fact that this was the first Indian study in which results from a control population tested for APC-R and deficiencies of AT, protein C and protein S were compared with commercial kit literature. We found that the mean levels were slightly higher for protein C and protein S but that the ranges for other parameters were similar to those of Caucasian populations (Table 1). We did not come across any published papers in which the normal Indian population’s levels of thrombophilia markers were mentioned.

## CONCLUSIONS

The prevalences of APC-R, protein C and protein S in controls were 4%, 0% and 2%; while in patients with venous thrombosis, the corresponding values were 17.9%, 7.7% and 6.6 %, and the differences in all of these were statistically significant.

LA, aCL and low AT levels was higher in patients with venous thrombosis, but did not attain statistical significance in these patients (P > 0.05). Testing for fibrinogen, in addition to well-recognized markers including APC-R, protein S and protein C, seems to be useful in young patients with primary venous thrombosis and in patients with a family history of thrombophilia. Larger Indian population need to be studied, in order to detect factor V Leiden and other mutations of APC-R.
